# Lack of spacing effects during piano learning

**DOI:** 10.1371/journal.pone.0182986

**Published:** 2017-08-11

**Authors:** Melody Wiseheart, Annalise A. D’Souza, Jacey Chae

**Affiliations:** 1 Department of Psychology, York University, Toronto, Ontario, Canada; 2 LaMarsh Centre for Child and Youth Research, York University, Toronto, Ontario, Canada; Tokai University, JAPAN

## Abstract

Spacing effects during retention of verbal information are easily obtained, and the effect size is large. Relatively little evidence exists on whether motor skill retention benefits from distributed practice, with even less evidence on complex motor skills. We taught a 17-note musical sequence on a piano to individuals without prior formal training. There were five lags between learning episodes: 0-, 1-, 5-, 10-, and 15-min. After a 5-min retention interval, participants’ performance was measured using three criteria: accuracy of note playing, consistency in pressure applied to the keys, and consistency in timing. No spacing effect was found, suggesting that the effect may not always be demonstrable for complex motor skills or non-verbal abilities (timing and motor skills). Additionally, we taught short phrases from five songs, using the same set of lags and retention interval, and did not find any spacing effect for accuracy of song reproduction. Our findings indicate that although the spacing effect is one of the most robust phenomena in the memory literature (as demonstrated by verbal learning studies), the effect may vary when considered in the novel realm of complex motor skills such as piano performance.

## Introduction

The spacing effect refers to a comparison of temporal lags between learning sessions, with no lag representing massed learning. In general, longer lags between learning episodes results in greater retention accuracy. Thus, the spacing effect has potential to reduce forgetting of learned content, which is a persistent concern in educational settings, at work, and at home—from memory of shopping lists to maintaining storyline continuity from episode to episode of a television show to recreational singing of songs. The vast majority of the spacing effect literature focuses on verbal learning, and less than 10% of the literature examines motor learning [1–2]. Almost no literature on music learning exists. Musical training involves learning of semantic sequences, such as melody lines, and precise control of amplitude and timing. Thus, music learning provides a domain in which we can compare usefulness of the spacing effect across a range of skills.

### Spacing effect theories

Major spacing effect theories focus on verbal learning. Most theories are hybrids of context variability [3–4] and other mechanisms [5–8]. These approaches capitalize on variations in cues that are present during learning of discrete items, with more cues and more variation in cues improving later recall. Contextual cues could be implicit, such as unintentional and unrecognized temporal changes in mood, or explicit, such as deliberately altering learning location to a different type of room.

Another mechanism used to explain spacing effects during verbal learning, study-phase retrieval [5,9–10], relies on adding strength to an existing memory trace, following successful retrieval of that existing trace, with greater strength when the trace is more difficult to retrieve. This theory allows predictions to be made around questions such as, “What happens if the memory trace is easier or harder to retrieve.” One of the major differences between context variability and study-phase retrieval is whether a single, very elaborate memory trace is formed versus many less elaborate and more independent memory traces. Theories that combine the two mechanisms exist [5].

Deliberate introduction of contextual interference decreases performance during motor skill acquisition and improves later retention [11]; however, too much contextual interference reduces retention performance [12], indicating that some degree of contextual interference is desirable, while too much is undesirable. Two theories have been developed to explain contextual interference effects: elaborative processing and action plan reconstruction [13–14]. Elaborative processing is based on changes in the number of inter- and intra-task comparisons, which can be manipulated by deliberately introducing additional tasks in working memory, such as through frequent task changes [15]. Action plan reconstruction is activated when a motor skill has left working memory, which requires recall of the action plan from long-term memory before the action can be performed [14]. A contextual interference theory that combines neural and behavioral data suggests a role for both elaboration and reconstruction processes [16].

Additional explanations for effects of spacing on motor skill performance improvements exist. One suggestion is that processes involved in task switching are involved—specially, processes involved in task set reconfiguration [17]. Another suggestion is that error processing mechanisms are involved [18], perhaps through alternation of tasks increasing the probability that error detection and correction occurs. Neither of these explanations has been explored in detail in empirical or theoretical spacing effect literature.

### Empirical spacing effect data

The spacing effect has a large impact on verbal recall, and an optimal lag exists between learning episodes [1,19–20]. In a typical study, there are two learning episodes with experimentally varied temporal lags between them, and a fixed retention interval at which test performance is measured. A meta-analysis demonstrates that the spacing effect persists despite changes in a large number of stimulus factors, including stimulus familiarity, complexity, form (e.g., words vs. pictures), cue relatedness, learning goals (i.e., intentional or incidental), and presentation context [21]. Estimates for the percentage of studies showing a spacing effect using verbal material range from 72% to 81%, and the average effect size is large, *d* = 0.85 [1–2].

In addition to an in depth meta-analysis of verbal learning [1], two meta-analyses have covered the spacing effect during motor learning. Lee and Genovese [22] found 73 effect sizes, with an average effect size of *d* = 0.79, a large effect. Donovan and Radosevich [23] found 40 effect sizes for motor learning tasks (their task clusters 1 and 3). The average effect size was *d* = 0.76, a large effect [23]. Moss [2] found that 89% of motor learning studies produced a spacing effect benefit, in line with the percentage of verbal learning studies that show a benefit from spacing.

Spacing effect and music learning studies show inconclusive results [24–28]. Simmons [26] measured accuracy with visible sheet music and found that fewer errors were made when learning took place with 24-hour vs. 5-min and 6-hour lags. However, the study did not include a retention interval, so we cannot assess retention benefits from spacing. Rubin-Rabson [25] asked musicians to learn songs at 1-hour and 2-day lags between learning episodes. The number of required relearning trials was determined after a two-week retention interval. Number of trials during relearning was allowed to vary, with a criterion of perfect performance at the end of relearning. It is likely that more relearning trials were required at longer lags, which confounds lag with amount of relearning. Thus, retention data cannot be interpreted. Cash [24] investigated the impact of a 5-min rest period during sequence learning, either early or late in the training sequence, and found that a gap early in learning improved retention the following day, relative to no rest period or a rest period late in practice. While not a standard spacing effect study, this study does suggest that the spacing effect will help memory for sequences.

Two interleaving and music learning studies exist. Interleaving is a comparison of blocked and mixed trial types, as opposed to a manipulation of lag, limiting interpretability in the context of the present research. Stambaugh and Demorest [28] studied accuracy and musicality of 8-bar pieces played on wind instruments (i.e., clarinet or saxophone), learned via massing or interleaving, after a one-day retention interval. They found a ceiling effect on accuracy, and no differences in musicality across conditions. Stambaugh [27] studied clarinet pieces learned via massing or interleaving, with a one-day retention interval, and failed to find accuracy effects. Again, accuracy analysis was complicated by performance approaching ceiling. Thus, no data appear to exist that allow us to determine if a spacing effect exists for music skill retention.

One study on music learning and timing exists. Stambaugh [27] studied clarinet pieces learned via massing or interleaving, with a one-day retention interval, and measured speed and evenness (i.e., timing). She failed to find evenness effects. She found that interleaving helped subjects sound notes more quickly, for a general speed benefit. There are no extant spacing effect and music learning studies that allow us to determine if a spacing effect exists for musical skills. Looking more broadly to the complex motor skill retention literature, only a handful of studies exist, and it is not clear whether spacing benefits retention of these skills, because the vast majority of the literature has examined acquisition of simple motor skills.

### Current study

Music learning is a complex skill that requires simultaneous use of many components, including reading visually presented musical notes, mentally connecting those notes to a relevant motor output and a desired sound, comparing auditory feedback to the desired sound, and adjusting motor output to decrease the discrepancy between actual and desired sounds. Our study combines a semantic component, memorizing a sequence of notes, and fine motor skill, moving fingers to press notes on a piano. Through our piano-playing tasks, we are able to separate

- (Task 1) Semantic–No Memory: a combination of real-time semantic verbal processing and procedural motor control, via accuracy of reproducing a musical sequence that does not require memorization (Hypothesis 1),
- (Task 1) Timing–No Memory: learning of temporal consistency, another continuous form of motor control, via consistency of keypress timing (Hypothesis 2), and
- (Task 1) Motor–No Memory: a continuous form of motor control, via ability to press keys with consistent amplitude force (Hypothesis 3),
- (Task 2) Semantic–Memory: a combination of semantic memorization and procedural motor control, via accuracy of reproducing a memorized sequence of notes that comprise a song (Hypotheses 4 and 5).

We are not aware of existing studies comparing these four abilities, within the spacing effect field or in other literatures. We chose not to include a standard verbal learning condition because a large number of verbal studies exist in the literature, and these additional data would not add to the field enough to justify increasing subjects’ fatigue.

No studies that we are aware of have examined whether there is an optimal lag between learning episodes during motor skill learning, in which longer lags increase performance, but if the lag becomes too long performance suffers. This effect is well established in the verbal learning literature, with improving accuracy as lag increases and then decreasing accuracy as lag further increases, at timescales from minutes [29] to a year [19–20]. Given forgetting rate in motor learning depends on task type [30–31], it is possible that different forms of motor skill will have different optimal lags.

Hypothesis 1: Longer lags will increase accuracy in Task 1.
Hypothesis 2: Longer lags will decrease *SD* of latency in Task 1.
Hypothesis 3: Longer lags will decrease *SD* of amplitude in Task 1.
Hypothesis 4a: Longer lags will increase accuracy in Task 2.
Hypothesis 4b: If longer lags increase accuracy in Task 2, an optimal lag will exist, such that accuracy will increase as lag increases and then decrease with further increases in lag.

## Materials and methods

### Subjects

A total of 100 individuals were run, approximately 20 per experimental condition. This sample size was chosen to provide 95% power, based on an estimated large effect size from previous verbal and motor learning spacing effect meta-analyses (*d* = 0.85, [1], verbal learning; *d* = 0.76, [23], motor learning; *d* = 0. 79, [22], motor learning). Subjects were university students enrolled in an introductory psychology course who were awarded course credit for participation. Almost all (n = 96) were right handed, all were free of physical limitations that would affect task performance (e.g., hearing problems, hand or motor disabilities), and most (n = 94) had never played piano. A fifth (n = 18) reported being able to read sheet music, and half (n = 47) reported having played a musical instrument at some point in their past. Individuals with some musical background were evenly distributed across conditions. This research was approved by the Human Participants Review Sub-Committee at York University, e2014-255. Data and protocols are available at https://osf.io/kehv9.

### Apparatus

#### Korg SP-250 88-key portable digital piano and Musical Instrument Digital Interface (MIDI) controller

The digital piano included weighted keys, and the included MIDI controller was connected to a MIDI to USB interface, which was connected to a Mac laptop. MIDI provides a real-time recording of note onset and offset (~1 ms accuracy), amplitude (128 gradations), note name (which key was pressed), and other information not relevant to this study. On the piano, the keys C3, D3, E3, F3, and G3 were labeled 5, 4, 3, 2, and 1, respectively, and the keys C4, D4, E4, F4, and G4 were labeled with their note name.

#### MIDI Monitor software

This open source software was used to display MIDI events, so the data could be recorded.

#### Metronome

This device produces clicks at a programmable speed. It was set to 66 beats per minute, which is typically perceived as a slow tempo.

### Materials

#### Sheet music

**Fig 1:**
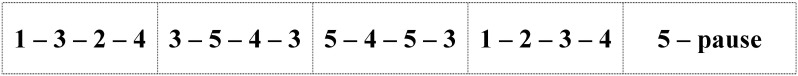
Note sequence played by the left hand. 5 (C3) = pinky and 1 (G3) = thumb.

A 17-note sequence of notes was placed on the piano during practice sessions (Fig 1). Typical sheet music notations were replaced with numbers. The sequence was selected from Hanon [32] (first eight notes from exercise #7 and remaining 9 notes modeled after exercise #4), which is a set of exercises that train pianists in speed, precision, agility, and strength of fingers. The Hanon exercises are ideal for measuring motor learning because they are specifically designed to target and improve motor ability. The selected sequence targeted the fourth and fifth fingers of the left hand, which have less strength than the other fingers, making playing evenly across fingers especially difficult.

#### Background questionnaire

This questionnaire included information on gender, handedness, age, university major, years of education, bilingualism, mother’s education, hearing and vision problems, medical background, history of musical training, and hobbies.

#### Happiness, comfort, and performance anxiety

Because anxiety is a potential confounding factor that could affect motor performance, a scale examined anxiety in several contexts. Two questions, “How happy are you right now?” and “How comfortable are you right now?,” were asked both at the beginning and end of the study to measure short-term changes in anxiety and comfort states. Overall anxiety traits were measured with twelve additional questions, such as “I have a fear of making mistakes.” at the end of the study.

### Design

There were five lag conditions (0-, 1-, 5-, 10-, and 15-min) and one retention interval (5-min). Lag was between-subjects, with random assignment to one of the five lag conditions. Task was within-subjects. Within a given subject, lag was the inverse between Task 1 and Task 2 (e.g., 0-min for task 1 and 15-min for task 2; see Table 1), to minimize overall session length.

**Table 1:** Demographic data: *M* (*SD*).

Task 1 Lag (min)	Task 2 Lag (min)	n	Age (years)	Gender (% female)	Mother’s Education (years)	Bilingualism^a^
15	0	20	19.6 (2.1)	83	13.7 (3.2)	80.4 (21.5)
10	1	20	19.6 (2.0)	68	13.6 (2.2)	73.3 (25.7)
5	5	19	21.3 (3.6)	65	12.2 (5.6)	71.9 (30.1)
1	10	20	19.7 (3.0)	70	13.4 (3.1)	72.2 (28.9)
0	15	21	19.9 (2.8)	81	14.7 (2.7)	61.0 (30.1)

^a^0 = fully monolingual, 100 = fully bilingual.

### Teacher-experimenters

There were two teacher-experimenters. One (JC) obtained the Associate of the Royal Conservatory diploma in piano, which is the highest academic credential awarded by the Royal Conservatory of Canada, and the other (AD) obtained the equivalent from the Associated Board of the Royal Schools of Music (ABRSM) in Britain. To further standardize the study, both teacher-experimenters used the same step-by-step teaching and testing instructions (i.e., protocol checklists) and coordinated with each other throughout data collection so that issues could be resolved in the same manner by each teacher-experimenter. Both ran pilot participants and debriefed subjects after the testing session was complete.

### Procedure

Subjects were welcomed to the study and given informed consent forms. They were given the opportunity to ask questions at any point in the study. They were shown how to sit with correct posture at a piano, and were given the opportunity to adjust seat position. The teacher-experimenter sat just behind them and to the left, so both could see the piano and the hand position. Subjects were shown how to place their left-hand fingers on the piano, and were instructed in proper hand technique when pressing keys. They were shown how the sheet music numbers corresponded to each key. They were given 30 s to practice pressing any of the labeled keys to the metronome, followed by 1-min of practicing pressing with different pressure levels.

#### Task 1: Motor skills

**Fig 2:**
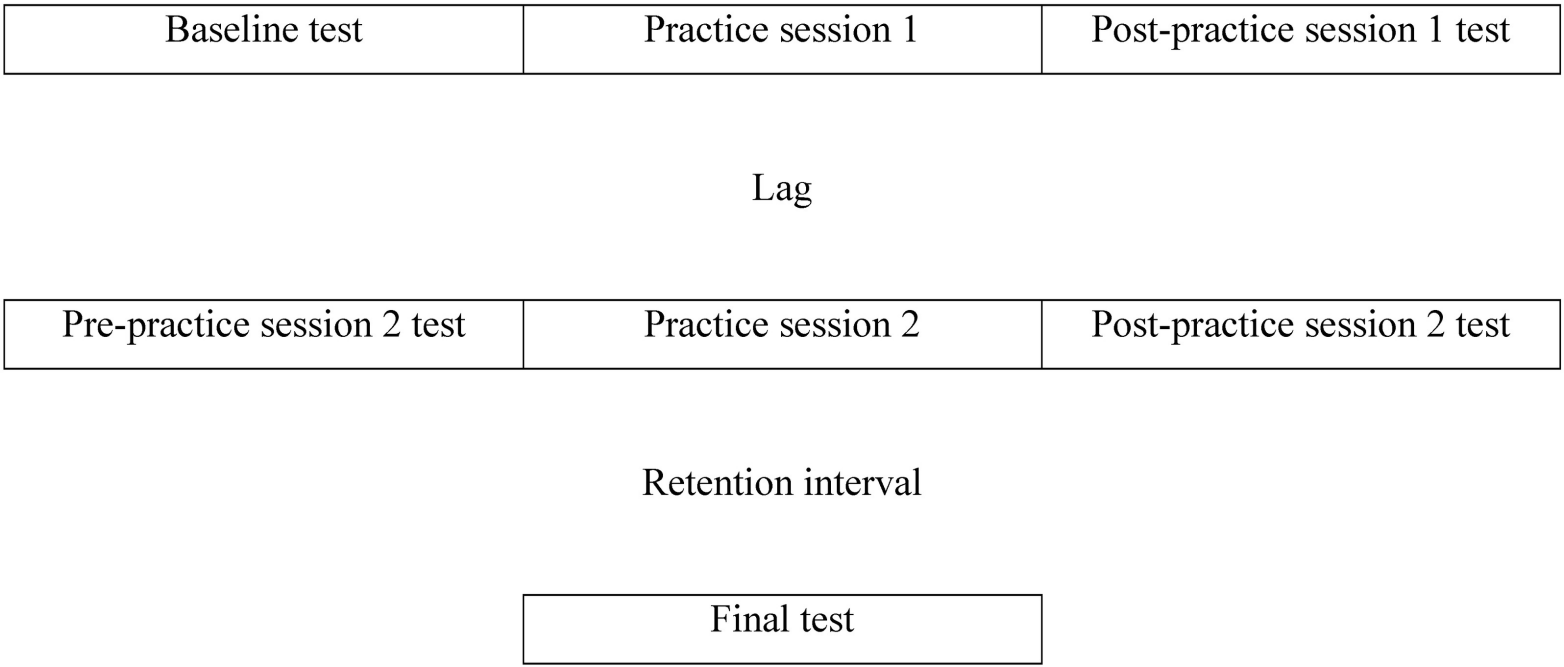
Study design.

Task 1 measured ability to learn note-finger mappings, utilize consistent finger pressure, and play using an accurate internal metronome. Subjects practiced a 17-note sequence (Fig 1) until they were able to correctly play all notes, using the metronome. Sheet music was visible on the piano throughout practice and tests. We gave feedback during the learning phase, but not during the testing phase, based on teacher-experimenter detection of errors. All tests consisted of the subject playing the sequence three times, without the metronome, from which note accuracy, note pressure, and note timing were measured. We used SD of amplitude and SD of latency as measures because a high degree of timing precision and finger pressure control is critical for a performance to be called musical (e.g., to demarcate note type [e.g., quarter, eighth] and time signature [e.g., 3/4, 4/4]). As a variance measure, SD captures the ability to be consistent at these two core skills. Music listening tests (e.g., Profile of Music Perception Skills [33]) are partially based on ability to detect variance in timing and amplitude. For each test, subjects were reminded to press keys as hard as possible while being even across all notes (i.e., we asked subjects to match the strength of their weakest finger, generally the pinky finger, so that loudness was matched across all notes), and to not correct mistakes. The task sequence was: baseline test, practice session 1, post practice session 1 test, lag, pre practice session 2 test, practice session 2, post practice session 2 test, retention interval, final test (Fig 2). Each practice session lasted for five min, during which subjects were reminded to apply as much pressure as possible while playing with even loudness, one note per metronome beat. During the lag and retention intervals, subjects colored, using crayons. Following the motor skills task, subjects completed background and performance anxiety questionnaires, followed by Task 2.

#### Task 2: Memorization

Task 2 measured ability to memorize short phrases from well-known songs. Subjects memorized the four to seven melody notes (i.e., one to two bars) associated with the title phrase of five songs: Row Row Row Your Boat, Old MacDonald Had a Farm, Oh Canada, Pop Goes the Weasel, and Happy Birthday (to You). All but eight subjects knew every song, and those eight knew at least three songs each, prior to starting the study. For this task, the sheet music used the letters C, D, E, F, and G, corresponding to labeled piano keys C4, D4, E4, F4, and G4. The task sequence was identical to that in Task 1.

For each song, in sequence, the teacher-experimenter played the song once, and then subjects played the tune five times with the sheet music visible, and then once without sheet music. Following song learning, subjects played each song five times with the sheet music, in sequence, which constituted a typical practice session. Then they were given tests on each song, in sequence, without sheet music, and they were instructed not to correct mistakes during the test phase (post practice session 1 test), which constituted a typical test session. Then a lag was given, during which subjects colored. Then a pre practice session 2 test was given, followed by a second practice session, and then a post practice session 2 test. Following the retention interval, a final test was given.

## Results

### Background data, happiness, comfort, and performance anxiety

We first ensured that groups were matched on demographic factors (Table 1), all *p*s > .28. We next examined happiness and comfort before and after the study and performance anxiety (Table 2). Performance anxiety was equivalent across groups, *F* < 1. In general, subjects were happier and more comfortable after the study compared to before, *F*(1,89) = 8.2, *p* = .005, η^2^_p_ = .084, which did not interact with lag group, *F* < 1; nor was there a main effect of lag group, *F* < 1.

**Table 2:** Happiness, comfort, and performance anxiety data: *M* (*SD*).

Task 1 Lag (min)	Task 2 Lag (min)	Happiness and Comfort Pre^a^	Happiness and Comfort Post^a^	Performance Anxiety^b^
15	0	84.6 (11.7)	84.6 (17.4)	5.6 (2.4)
10	1	78.8 (12.4)	85.1 (13.6)	5.6 (2.1)
5	5	79.7 (11.5)	82.0 (14.2)	5.8 (2.1)
1	10	75.9 (19.0)	80.3 (16.0)	5.6 (1.8)
0	15	73.9 (20.1)	82.8 (14.8)	5.0 (2.5)

^a^0 = low, 100 = high

^b^0 = low, 10 = high.

### Task 1: Motor skills

#### Data trimming

Within-subject, within-condition iterative trimming with a 3 SD cut-off was performed on *SD* of latency and *SD* of amplitude data. A total of 0.4% of cells were missing data, following computation of means, solely due to experimenter error saving data (i.e., the cache used for collecting MIDI data was not large enough for some subjects, and thus some pre-P1 trials were lost). Multiple imputation was used to fill in these scant missing cells.

#### Learning and forgetting analyses

Analyses were run to determine whether learning occurred during the practice phases, and whether forgetting took place during the lag. Accuracy reflects ability to play the note sequence correctly with the sheet music present (Table 3), which reflects ability to correctly translate notes that are read visually to motoric finger presses. *SD* of latency (Table 4) reflects ability to create an internal metronome, and then make finger presses in time to that metronome. *SD* of amplitude (Table 5) reflects ability to hear loudness differences and make small alterations to finger pressure to ensure that all loudness measurements are equal. Mean amplitude was 55 out of 127, neither at floor nor ceiling. Mean latency was 850 ms, slightly faster than the metronome latency of 909 ms (i.e., 70.6 beats per minute for subjects vs. 66 beats per minute as metronome speed).

**Table 3:** Percent correct on each task 1 test: *M* (*SD*).

Lag (min)	Baseline	Post Practice Session 1 Test	Pre Practice Session 2 Test	Post Practice Session 2 Test	Final Test
0	71.4 (20.2)	80.9 (18.9)	78.9 (15.7)	74.0 (18.3)	75.4 (16.4)
1	65.4 (17.9)	77.2 (17.8)	82.9 (16.6)	78.8 (17.3)	78.9 (20.5)
5	65.0 (27.8)	78.3 (20.0)	74.8 (23.2)	76.7 (20.7)	79.9 (19.2)
10	75.5 (18.9)	77.5 (17.2)	80.4 (17.6)	79.9 (20.3)	85.8 (16.5)
15	67.8 (21.4)	72.3 (19.4)	79.6 (25.7)	81.9 (22.1)	89.4 (11.7)

**Table 4:** SD of latency (ms) on each task 1 test: *M* (*SD*).

Lag (min)	Baseline	Post Practice Session 1 Test	Pre Practice Session 2 Test	Post Practice Session 2 Test	Final Test
0	237.9 (163.0)	110.2 (78.3)	109.3 (65.7)	112.3 (112.1)	71.5 (48.4)
1	194.4 (118.4)	127.4 (103.7)	106.2 (106.6)	99.1 (84.0)	99.6 (97.9)
5	276.6 (217.6)	143.1 (139.1)	174.5 (189.7)	123.8 (108.8)	98.7 (79.3)
10	169.3 (152.1)	110.8 (93.7)	98.4 (88.4)	90.6 (79.1)	91.1 (77.6)
15	180.4 (122.2)	126.1 (91.2)	114.8 (88.2)	97.0 (54.6)	79.2 (49.9)

**Table 5:** *SD* of amplitude on each task 1 test: *M* (*SD*).

Lag (min)	Baseline	Post Practice Session 1 Test	Pre Practice Session 2 Test	Post Practice Session 2 Test	Final Test
0	6.7 (2.4)	5.7 (2.2)	6.3 (1.9)	6.5 (2.9)	6.2 (3.4)
1	6.7 (2.2)	6.0 (1.8)	6.2 (1.9)	6.3 (2.5)	6.4 (1.9)
5	9.8 (5.9)	7.4 (3.2)	6.9 (3.9)	6.8 (2.5)	6.1 (2.2)
10	5.8 (2.7)	6.1 (2.9)	5.6 (1.9)	5.5 (2.3)	5.7 (2.2)
15	6.94 (2.1)	6.6 (2.2)	5.6 (1.5)	5.9 (2.2)	5.8 (2.4)

Baseline performance was examined to ensure that all groups were equivalent prior to the study, using a one-way between-subjects ANOVA with lag as a factor. Baseline test performance was compared to post practice session 1 test performance, to assess initial learning, using a two-way mixed ANOVA with time (baseline or post) and lag as factors. Post practice session 1 test performance was compared to pre practice session 2 test performance, to assess forgetting across the lag, using a two-way mixed ANOVA with time (post or pre) and lag as factors. Pre practice session 2 test performance was compared to post practice session 2 test performance, to assess relearning, using a two-way mixed ANOVA with time (pre or post) and lag as factors.

First, we examined accuracy. All groups were equivalent at baseline, *F* < 1. All groups improved with practice to an equal extent, *F*(1,95) = 12.2, *p* = .001, η^2^_p_ = .114, for the main effect of time during practice 1. No forgetting took place during the lag, *F*s < 1. No additional learning took place during the second practice session, all *F*s < 1.

Next, we examined *SD* of latency. All groups were equivalent at baseline, *F*(4,95) = 1.6, *p* = .188. Learning took place during practice session 1, shown by the main effect of time, *F*(1,95) = 60.9, *p* < .001, η^2^_p_ = .391. No forgetting took place during the lag, all *p*s > .28, and practice session 2 did not produce additional learning, all *p*s > .06.

Finally, we examined *SD* of amplitude. Groups were not matched at baseline, *F*(4,95) = 4.1, *p* = .004, η^2^_p_ = .117, which Tukey HSD tests showed was due to the lag-5 group having greater SD of amplitude than the other groups, which did not show SD differences. Practice 1 reduced *SD* of amplitude, *F*(1,95) = 9.5, *p* = .003, η^2^_p_ = .091, for the main effect of time. Unexpectedly, there was a main effect of lag, *F*(4,95) = 3.2, *p* = .018, η^2^_p_ = .117, and an interaction between lag and time, *F*(4,95) = 2.8, *p* = .03, η^2^_p_ = .105, neither of which should have occurred prior to the intervention. No forgetting took place during the lag, all *p*s > .07. No additional learning took place during the second practice session, all *p*s > .28.

### Spacing analyses

A one-way between-subjects ANOVA revealed that for accuracy, the lag effect was not significant, *F*(4, 95) = 2.24, *p* = .07, ŋ^2^ = .09. Thus, Hypothesis 1 was not supported. The planned contrast (K Matrix) between lag-0 and the combination of lags 1, 5, 10, and 15 was not significant, *F*(1, 95) = 3.63, *p =* .06, ŋ^2^ = .04 (Table 3).

For *SD* of latency, a one-way between-subjects ANOVA was run. There was no difference in *SD* of latency across lag conditions, *F* (4, 95) = .57, *p* = .68, ŋ^2^ = .02. Thus, Hypothesis 2 was not supported. The planned contrast (K Matrix) between lag-0 and the combination of lags 1, 5, 10, and 15 was not significant, *F*(1, 95) = 1.29, *p =* .26, ŋ^2^ = .01 (Table 4).

For *SD* of amplitude, a one-way between-subjects ANOVA was run. There was no difference in *SD* of amplitude across lag conditions, *F* (4, 95) = .28, *p* = .90, ŋ^2^ = .01. Thus, Hypothesis 3 was not supported. The planned contrast (K Matrix) between lag-0 and the combination of lags 1, 5, 10, and 15 was not significant, *F*(1, 95) = .18, *p =* .67, ŋ^2^ = .01 (Table 5).

### Task 2: Memorization

#### Data trimming

Occasionally, the teacher-experimenter failed to record the subject’s start cue on one or more trials, which caused missing data. Consequently, sample size was not equal across conditions. Specifically, n = 20 in all cells in the lag-0 and lag-15 conditions, n = 19 or 20 in the lag-1 condition, and n = 16 or 17 in the lag 5 and lag-10 conditions. No data trimming or imputation were used. Data were collapsed across the five learned songs. Only accuracy data were analyzed, as notes were of different duration, and participants were specifically instructed to focus on accuracy and not attend to loudness.

#### Learning and forgetting analyses

Analyses were run to determine whether learning occurred during the practice phases, and whether forgetting took place during the lag. Accuracy reflects ability to play the note sequence correctly with the sheet music not visible (Table 6), which represents memorization of the songs. The baseline test was performed one song at a time, with testing immediately following learning. All other tests were performed with memory for all five songs sequentially in a single testing period, and there was a time period between practice with the sheet music and the test, as well as an opportunity for interference between songs. Thus, the baseline test cannot be directly compared to the other tests.

**Table 6:** Percent correct on each task 2 test: *M* (*SD*).

Lag (min)	Baseline	Post Practice Session 1 Test	Pre Practice Session 2 Test	Post Practice Session 2 Test	Final Test
0	79.8 (18.8)	45.9 (23.1)	39.3 (20.8)	57.0 (26.0)	61.3 (24.3)
1	87.2 (18.3)	38.9 (22.3)	46.0 (21.3)	63.9 (24.7)	60.8 (24.7)
5	78.6 (23.4)	44.2 (25.9)	45.1 (24.0)	59.3 (24.6)	59.9 (22.5)
10	84.7 (19.4)	52.5 (25.2)	45.8 (23.2)	67.5 (23.6)	69.9 (26.2)
15	84.8 (21.5)	41.7 (20.0)	43.1 (19.9)	62.4 (21.9)	66.7 (23.0)

Baseline is not directly comparable to other tests due to testing method differences.

Baseline performance was examined to ensure that all groups were equivalent prior to the study, using a one-way between-subjects ANOVA with lag as a factor, and post practice session 1 performance were similarly analyzed as a second form of baseline equivalency check. Post practice session 1 test performance was compared to pre practice session 2 test performance, to assess forgetting across the lag, using a two-way mixed ANOVA with time (post or pre) and lag as factors. Pre practice session 2 test performance was compared to post practice session 2 test performance, to assess relearning, using a two-way mixed ANOVA with time (pre or post) and lag as factors.

All groups were equivalent at baseline and post practice session 1, *F*s < 1. No forgetting took place during the lag, *F* < 1. Learning took place during the second practice session, *F*(1,87) = 68.0, *p* < .001 η^2^_p_ = .439. No lag main effects or interactions were found for any analyses, all *F*s < 1.9.

#### Spacing analyses

A one-way between-subjects ANOVA revealed that the lag effect was not significant, *F* < 1. Nor was the planned contrast (K Matrix) between lag-0 and the combination of lags 1, 5, 10, and 15 significant, *F* < 1 (Table 3). Thus, Hypothesis 4 was not supported, and Hypothesis 5 was not testable.

## Discussion

We tested efficacy of the spacing effect for two forms of semantic music learning. One form required memorization of phrases and the other only required simple mapping of visually presented notes to fingers. We simultaneously measured timing and loudness during a complex motor task: mapping of an internal clock to finger pressing (SD of latency), and mapping of perceived loudness level to finger pressing (SD of amplitude). No condition showed a spacing effect during acquisition or retention, even though a substantial degree of learning occurred during practice sessions. Relearning picked up at exactly the same point where learning ended in the previous practice session, with no forgetting, in line with some other studies of complex motor skill learning [34–35]. These findings offer valuable insights on the extent and limitations of the spacing effect via the novel context of complex motor skills, since the majority of previous research has focused on verbal learning, with a smaller proportion on simple motor skills.

It may be that the spacing effect would have been evident had forgetting occurred. Because our lags were all 15 min or less, and retention interval was 5 min, there was little opportunity to forget the semantic information that was presented. Instead, we found no evidence of forgetting, which is a key element in study-phase retrieval theory. Our results suggest that longer lags and retention intervals should be tested, so that forgetting is more likely to occur. Verbal learning studies that produce robust spacing effects often involve a great deal of forgetting during the lag [19–20]. We located one study that produced forgetting in a spaced condition but not in a massed condition [36], and no evidence to the contrary. Notably, Dail and Christina [36] showed a spacing effect for both motor skill acquisition and retention. Thus, we tentatively propose that a spacing effect should be found whenever forgetting occurs during a spaced condition but not in a massed condition.

Two of our measures, amplitude and latency, involved changes in variance rather than changes in mean performance. We were unable to locate many studies that examined variance data. At least two studies suggest that this type of measure, which does not have a discrete correct answer and that does not always result in perfect or best possible performance at an asymptote, has potential to demonstrate benefits in retention as a consequence of a lag manipulation [37–38]. Thus, our failure to find a spacing effect for SD of latency and SD of amplitude measures does not appear to be a consequence of the variance aspect of those outcome measures.

In a review of the spacing effect across a wide range of topic areas, we found mixed results for motor skill acquisition and retention. We found it impossible to determine if and when a given motor skill manipulation would produce a spacing effect, and we were unable to determine factors that led to null, mixed, and positive spacing effect benefits, due to the complexity of existing data, task variability, methodological confounds, incomplete reporting of data, and a paucity of designs intended to test theoretically meaningful factors. Previous meta-analyses suggest that easier motor tasks might be more likely to produce a spacing effect, although few studies examine retention, and it is quite challenging to compare across studies due to major differences in methodology (e.g., vastly different lags and retention intervals; vastly different task types and task difficulty). Similarly, other reviewers of this literature have found it challenging to predict when and why a spacing effect will occur [22–23].

Researchers need to systematically explore different types of situations and lags in relation to each other, as we did in the present study by combining, semantic, amplitude, and timing measures, and through our use of multiple lags to examine whether an optimal lag exists. Only with a sufficiency of data will future reviewers be able determining when and why a spacing effect will exist during motor learning. Given the variability of observed effects in motor learning, the link between specific study conditions and a spacing effect should be systematically examined to conclude the generalizability of such an effect. In the current study, we observed no spacing benefits on a 17-note musical sequence in piano novices with five lags between learning episodes (0-, 1-, 5-, 10-, and 15-min), a 5-min retention interval, and three measurements (accuracy of note playing, consistency in pressure applied to the keys, and consistency in timing).

Some subjects had previous musical exposure, such as playing an instrument or ability to read sheet music. Because these subjects were evenly distributed across conditions, they will not have affected the pattern of data. Prior training might have resulted in less room for improvement over the course of the experiment in our measures. Alternatively, it may have lead to a better ability to attend to the instructions and to focus on improving performance (due to previous practice with the techniques used). Our sample size was not large enough to test this possibility.

### Future directions

We failed to find spacing effect benefits to three aspects of music learning: semantic (i.e., accuracy of reproduction of a sequence of notes), consistency of finger pressure (i.e., loudness), and consistency of timing. Taken with the variability of existing findings across a range of tasks, the field would benefit from additional studies that systematically manipulate factors that might cause the spacing effect to appear during these forms of learning, outside the traditional verbal domain of word learning. Unless studies are able to produce forgetting, it is possible that spacing effect benefits will not occur.
